# Supplementary material for: FcγRIIb differentially regulates pre-immune and germinal center B cell tolerance in mouse and human
Source: Nat Commun. 2019 Apr 29;10:1970. doi: 10.1038/s41467-019-09434-0 (PMC6488660; doi:10.1038/s41467-019-09434-0)
Supplement: Supplementary file 1 — Supplementary Information [file 41467_2019_9434_MOESM1_ESM.pdf]

Supplementary Information

FcγRIIb differentially regulates pre-immune and germinal center B cell tolerance in mouse and human.

Espéli *et al.*

Supplementary Figure 1: Reduced FcγRIIb expression is associated with reduced number of autoreactive B cells in the BM and the spleen.

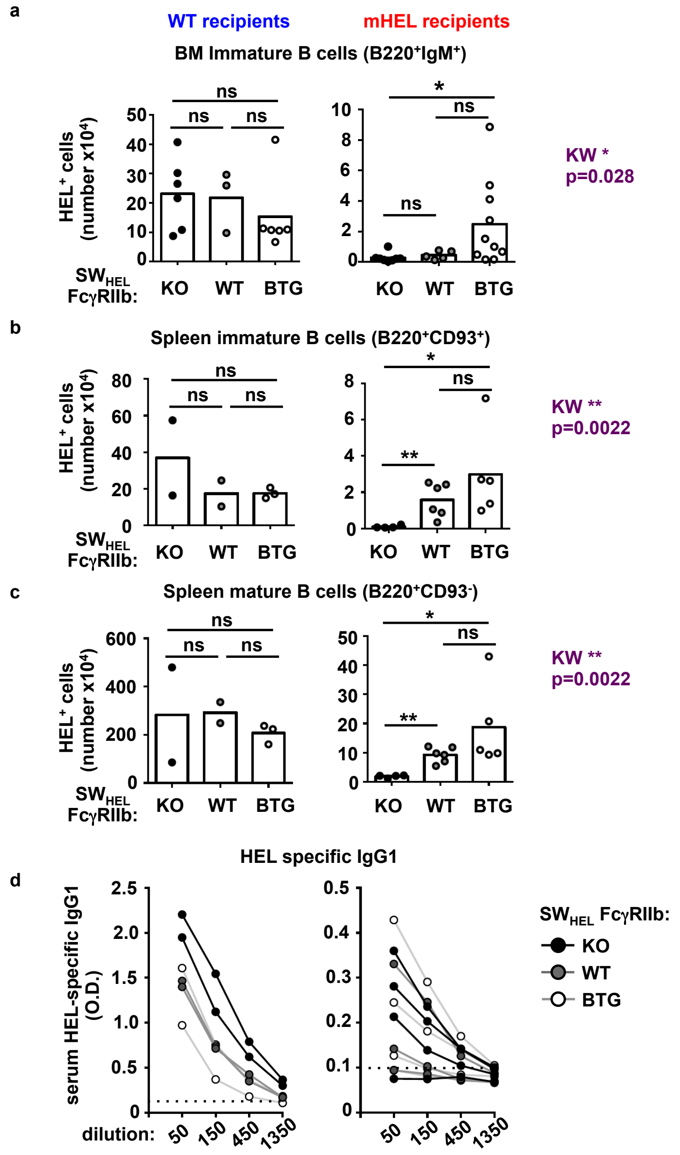

(**a-c**) Quantification of the absolute number of HEL-specific BM immature B cells (**a**), splenic immature B cells (**b**) and splenic mature B cell (**c**) in WT (left panel) and mHEL (right panel) recipient mice. The three cell populations were gated as in figures 1 and 2. For panel **a**, two pooled experiments are shown (mHEL recipients: n=5-10 mice per group; wt recipients: n=3-6 mice per group). For panels **(b-c)** one experiment representative of 5 is shown (mHEL recipients: n=4-6 mice per group; wt recipients: n=2-3 mice per group). (**d**) Serum HEL-specific IgG1 titer in unimmunized WT (left panel) and mHEL (right panel) recipient mice. The optical density (O.D.) of the blank, indicating the threshold of detection, is indicated by a dotted line. WT recipients: n=2-4 mice per group; mHEL recipients: n=3-4 mice per group. The mean is represented and each dot corresponds to an individual mouse. The p-values were determined with the Kruskal-Wallis (KW) non-parametric test when comparing the 3 groups (KW: the exact p-value is indicated in purple for the mHEL recipient. All KW tests were not significant for the WT recipients) and with the Mann-Whitney non-parametric test when two conditions were compared (indicated by black stars). *: $p<0.05$; **: $p<0.01$. Comparisons generating non-significant p-values were indicated with “ns”. Source data are provided as a Source Data file.

Supplementary Figure 2: Reduced FcγRIIb expression increases deletion in the BM and the spleen in mice constitutively expressing HEL as an autoantigen.

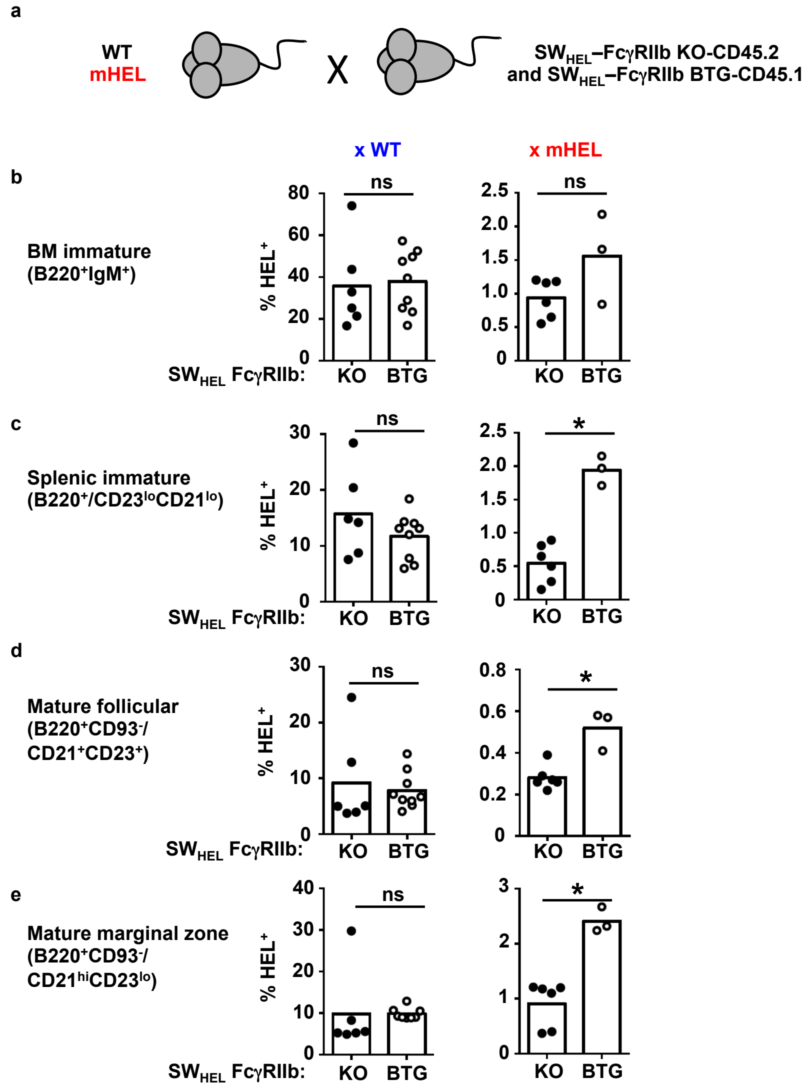

(**a**) Experimental procedure: SW_{HEL}-FcγRIIb KO-CD45.2 and SW_{HEL}-FcγRIIb BFcR-CD45.1 mice were crossed with mHEL mice. Mice bearing the SW_{HEL} transgenes and one (x mHEL) or no (x WT) allele of the mHEL transgene were analyzed. (**b-e**) Quantification of the frequency of HEL-specific bone marrow immature B cells (**b**), splenic immature B cells (**c**), splenic mature follicular B cells (**d**) and splenic mature marginal zone B cells (**e**) in WT (left panels) and mHEL (right panels) mice. The different populations were gated as in Figure 1 and Figure 2. x mHEL: n=3-6 mice per group; x WT: n=6-9 mice per group. Two pooled experiments are shown. The mean is represented and each dot corresponds to an individual mouse. The p-values were determined with the Mann-Whitney unpaired two-tailed test. *: $p < 0.05$,. Comparisons generating non-significant p-values were indicated with “ns”. Source data are provided as a Source Data file.

Supplementary Figure 3: Immunization does not modify the effect of FcγRIIb on B cell pre-immune tolerance.

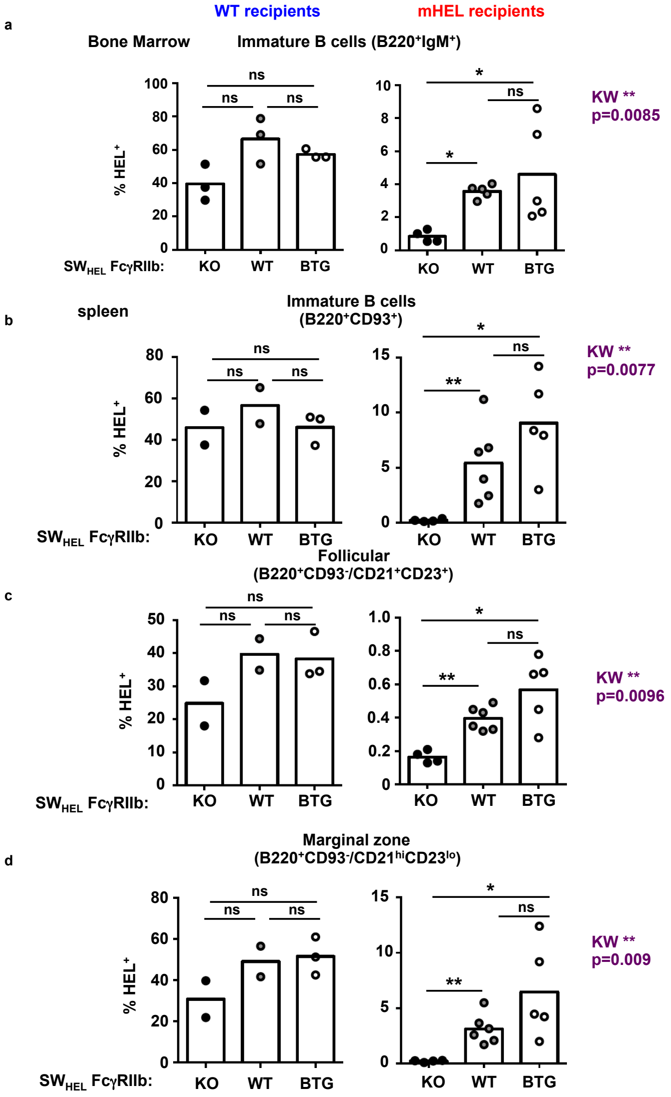

(**a-d**) Wild type and mHEL recipients (both CD45.2) were irradiated and reconstituted with bone marrow from SW_{HEL}-FcγRIIb KO-CD45.2, SW_{HEL}-FcγRIIb WT-CD45.1 or SW_{HEL}-FcγRIIb BFcR-CD45.1 donor mice and immunized post-reconstitution with SRBC-HEL. The different populations were gated as in Figure 1 and 2. Quantification of the frequency of HEL-specific bone marrow immature B cells (**a**), splenic immature B cells (**b**), splenic mature follicular B cells (**c**) and splenic mature marginal zone B cells (**d**) in WT (left panel) and mHEL (right panel) recipient mice. One experiment representative of 3 is shown. Wild-type recipients: n=2-3 mice per group; mHEL recipients: n=4-6 mice per group. The mean is represented and each dot corresponds to an individual mouse. The p-values were determined with the Kruskal-Wallis (KW) non-parametric test when comparing the 3 groups (KW: the exact p-value is indicated in purple for the mHEL recipient. All KW tests were not significant for the WT recipients) and with the Mann-Whitney non-parametric test when two conditions were compared (indicated by black stars, all p-values for the WT recipients were non significant). *: $p<0.05$; **: $p<0.01$. Comparisons generating non-significant p-values were indicated with “ns”. Source data are provided as a Source Data file.

Supplementary Figure 4: The frequency of T_{FH} and T_{FR} was not affected by FcγRIIb expression after SRBC-HEL immunization.

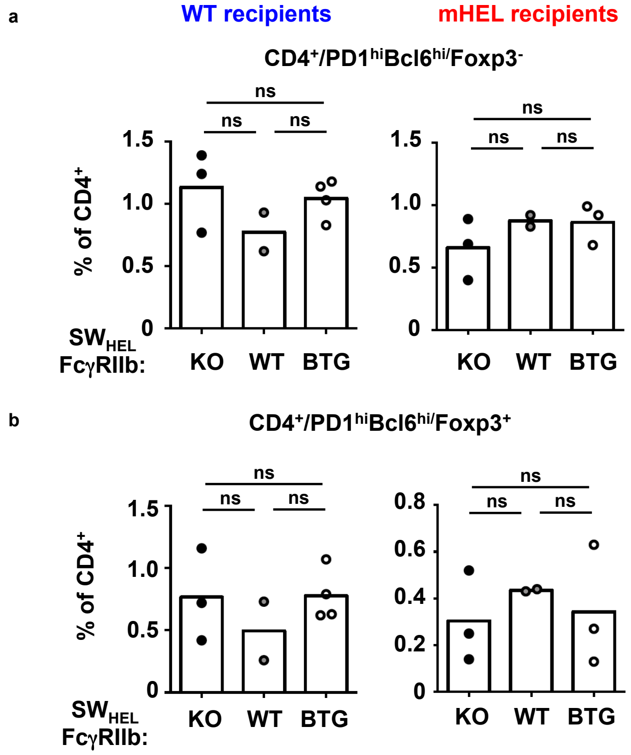

(**a-b**) Quantification of the frequency of T follicular helper (T_{FH}; $CD4^+/PD1^{hi}Bcl6^{hi}/Foxp3^-$) (**a**) and T follicular regulatory (T_{FR}; $CD4^+/PD1^{hi}Bcl6^{hi}/Foxp3^+$) (**b**) cells in WT (left panels) and mHEL (right panels) mice after SRBC-HEL immunization. Wild-type recipients: n=2-4 mice per group; mHEL recipients: n=2-3 mice per group. The mean is represented and each dot corresponds to an individual mouse. The p-values were determined with the Kruskal-Wallis (KW) non-parametric test when comparing the 3 groups and with the Mann-Whitney non-parametric test when two conditions were compared. All p-values were non significant. Source data are provided as a Source Data file.

Supplementary Figure 5: The antibody forming cell spot size and intensity are not affected by FcγRIIb expression after SRBC-HEL immunization.

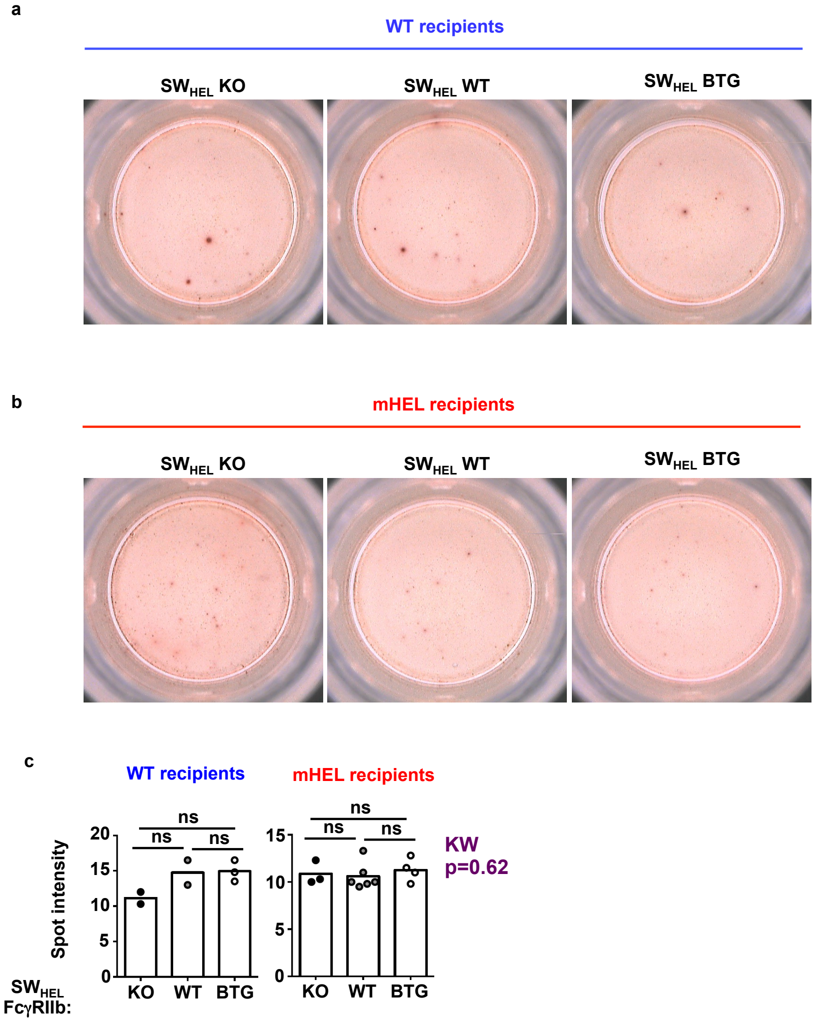

(**a-b**) Representative ELISpot wells of IgG1 HEL-specific antibody forming cells (AFCs) in the spleen of SRBC-HEL immunized WT (**a**) and mHEL (**b**) recipient mice. (**c**) Spot intensity is shown for the six experimental groups. For all panels one experiment representative of at least 2 is shown. Wild-type recipients: n=2-3 mice per group; mHEL recipients: n=3-6 mice per group. The mean is represented and each dot corresponds to an individual mouse. The p-values were determined with the Kruskal-Wallis (KW) non-parametric test when comparing the 3 groups (KW: the exact p-value is indicated in purple for the mHEL recipient. All KW tests were not significant for the WT recipients) and with the Mann-Whitney non-parametric test when two conditions were compared. All p-values were non significant. Source data are provided as a Source Data file.

Supplementary Figure 6: Natural polymorphisms of *Fcgr2b* have an opposing impact on pre- and post-immune tolerance checkpoints.

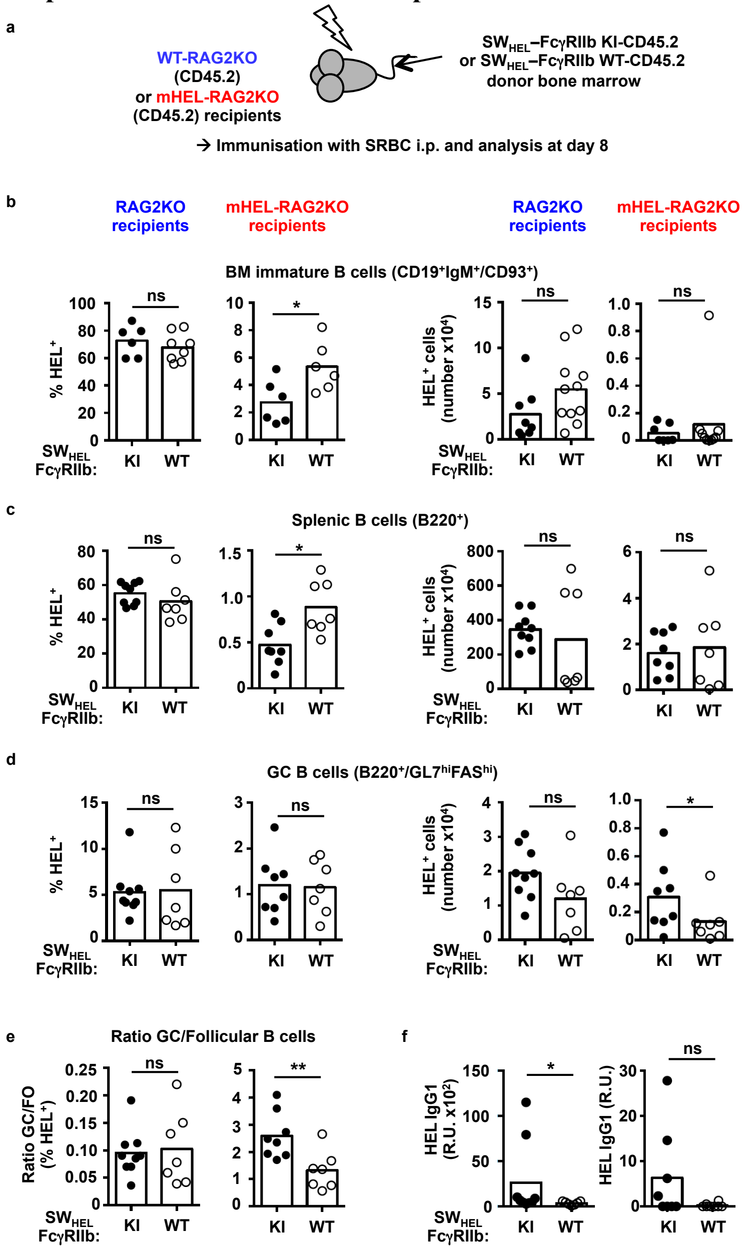

(**a**) Experimental procedure: RAG2KO and RAG2KO-mHEL recipients (both CD45.2) were irradiated and reconstituted with bone marrow from SW_{HEL}-FcγRIIb$^{wild/H1}$ KI-CD45.2 or SW_{HEL}-FcγRIIb WT-CD45.2 donor mice. Post reconstitution, chimeras were immunized with SRBC intraperitoneally and analyzed 8 days later. All populations were gated as in Figures 1, 2 and 4. (**b-d**) Quantification of the frequency (left panels) and absolute number (right panels) of HEL-specific B cells in WT-RAG2 KO (left panel) and mHEL-RAG2 KO (right panel) recipient mice: BM immature B cells (**b**), splenic B cells (**c**), splenic GC B cells (**d**). **(e)** Ratio of HEL specific GC B cells and total B cells in WT-RAG2 KO (left panel) and mHEL-RAG2 KO recipient mice (right panel). (**f**) Serum HEL-specific IgG1 titers in RAG2KO (left panel) and RAG2KO-mHEL (right panel) recipient mice. Two experiments were pooled. RAG2KO recipients: n=8-9 mice per group; RAG2KO-mHEL recipients: n=7-8 mice per group. The mean is represented and each dot corresponds to an individual mouse. The p-values were determined with the unpaired two-tailed Mann-Whitney non-parametric test. ns: non significant; *: $p<0.05$; **: $p<0.01$. Source data are provided as a Source Data file.

Supplementary Figure 7: Summary of the flow sorting strategy for healthy *FCGR2B-232* genotyped donor PBMC B-cell subsets.

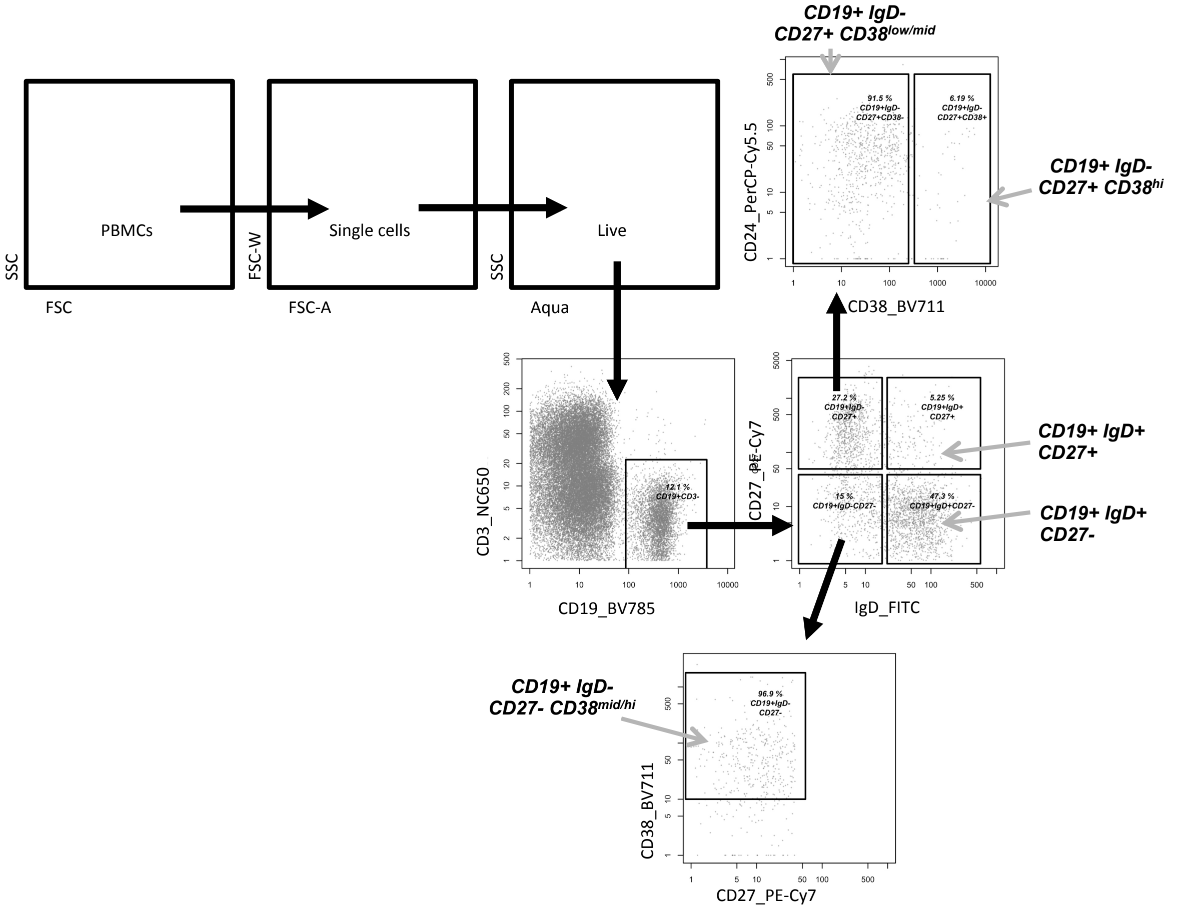

Human PBMCs were stained and cell sorted based on the strategy represented. Leukocytes were first gated, then doublets were excluded and only live cells were considered for further sorting. B cells were gated as $CD19^+$ and $CD3^-$. B cells were then divided based on the expression of IgD, CD27 and CD38. $CD19^+/IgD^+CD27^-$, $CD19^+/IgD^+CD27^+$, $CD19^+/IgD^-CD27^-/CD38^{mid/hi}$, $CD19^+/CD27^+IgD^-/CD38^{low/mid}$ and $CD19^+/CD27^+IgD^-/CD38^{hi}$ cells were sorted for repertoire analysis. CD38 positivity was determined based on an unstained control.

Supplementary Figure 8: Schematic diagram of the *IGH* sequencing strategy.

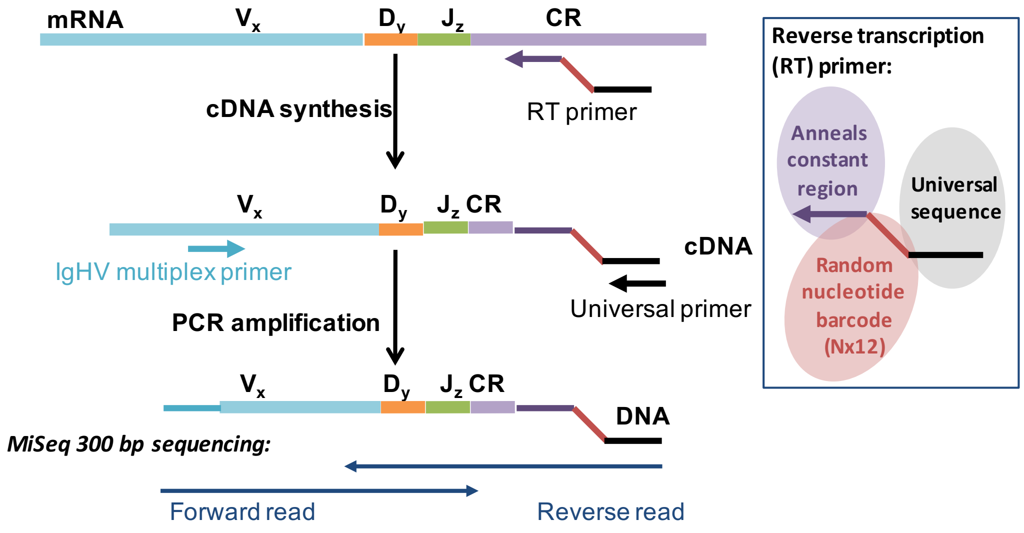

In the reverse transcription (RT) step, the primer anneals to the constant region of the B-cell receptor mRNA to generate cDNA with a random 12 nucleotide barcode. This barcode can be computationally used to reduce PCR amplification biases after sequencing. The product is cleaned and PCR amplified using multiple primers binding to the FR1 region of the *IGHV* genes along with a universal sequence complementary to the end of the RT primer.

Supplementary Figure 9: B-cell subset distribution is not altered by the *FCGR2B-I/T232* genotypes.

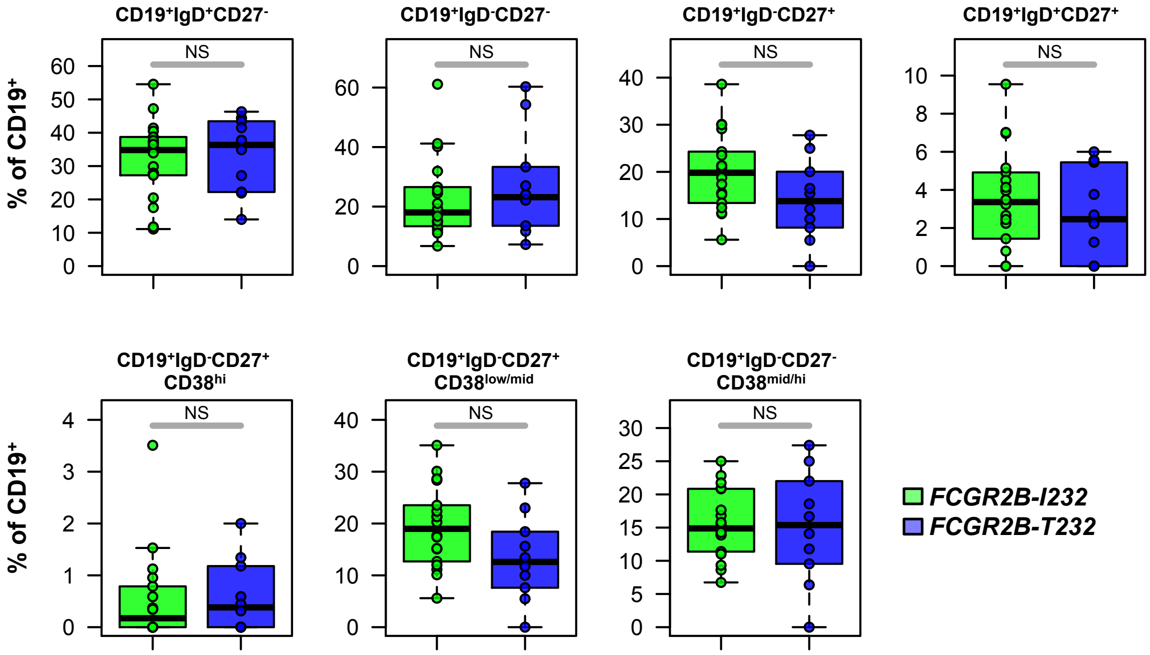

Boxplots of the proportion of each B-cell subset defined in Supplementary Figure 7 as a proportion of $CD19^+$ B-cells, where blue and green represent healthy donors with the *FCGR2B-T232* and *FCGR2B-I232* genotypes respectively.

Supplementary Figure 10: Distribution of isotype usage is not altered by the *FCGR2B-I/T232* genotypes.

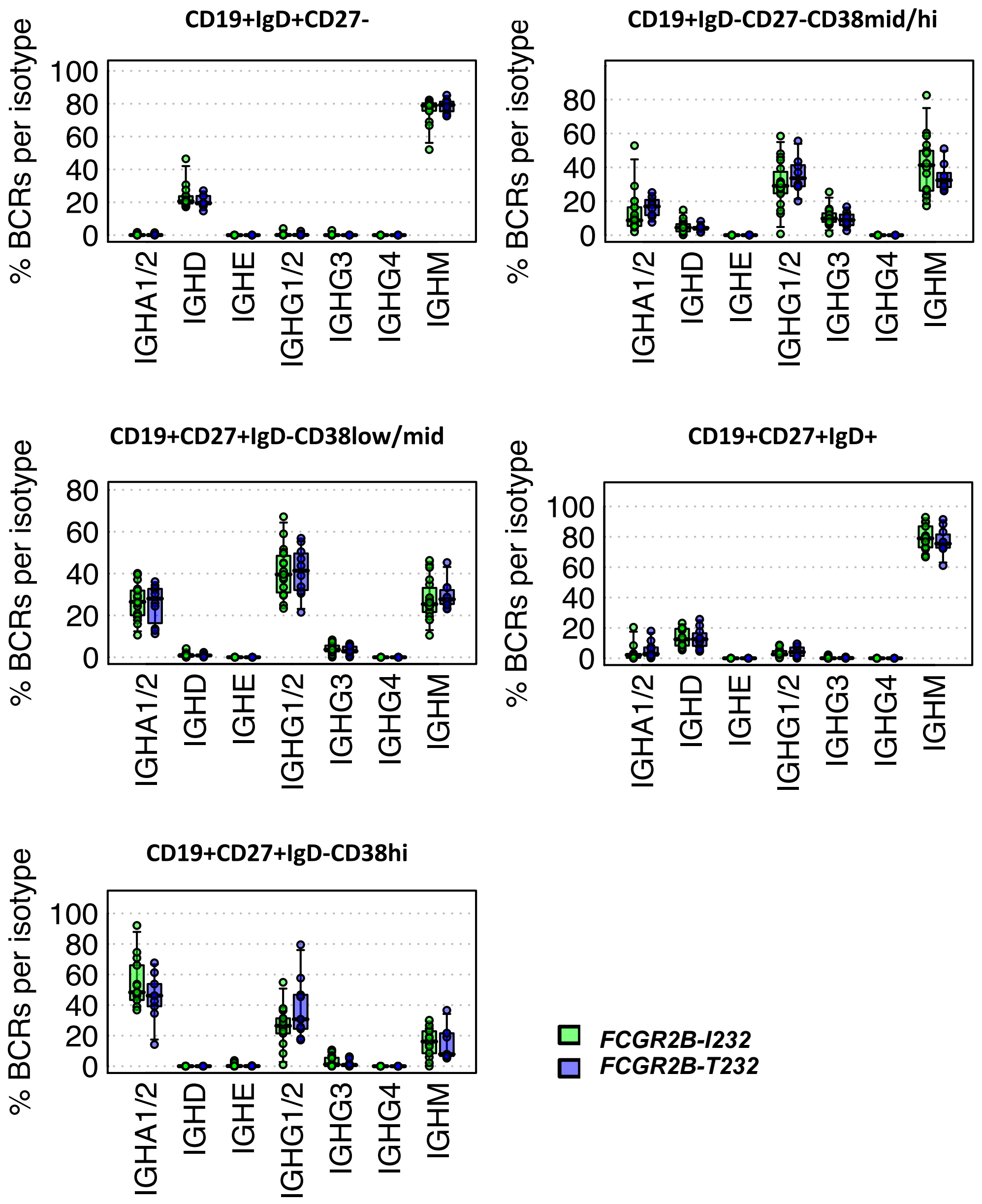

Distribution of isotype usage as a percentage of total *IGH* reads across cell sorted B-cell subsets between healthy *FCGR2B-232* genotyped donors. Blue and green represent healthy donors with the *FCGR2B-T232* and *FCGR2B-I232* genotypes respectively.

Supplementary Table 1: Table of *IGH* sequencing samples and number of *IGH* reads after filtering.

Age at visit (years)	*Genotype*	*B-cell population*	*Individual*	*Number of reads with barcodes*	*Number of unique RNA IGH molecules sequenced (after filtering)*	*Number of unique IGH sequences*
65	TT	CD19+CD27+IgD-CD38lo/mid	S14000001A	33222	20862	10291
61	TT	CD19+CD27+IgD-CD38 lo/mid	S14000004D	30257	13546	5032
52	TT	CD19+CD27+IgD-CD38 lo/mid	S14000005E	29513	19493	10060
65	TT	CD19+CD27+IgD-CD38 lo/mid	S14000006F	32047	22575	11331
52	TT	CD19+CD27+IgD-CD38 lo/mid	S14000008H	57202	27130	15388
52	TT	CD19+CD27+IgD-CD38 lo/mid	S14000010K	46426	26286	13800
77	TT	CD19+CD27+IgD-CD38 lo/mid	S14000012M	39488	30805	15038
54	TT	CD19+CD27+IgD-CD38 lo/mid	S14000013N	39851	14992	6421
65	TT	CD19+CD27+IgD-CD38 lo/mid	S14000014P	49548	29871	15472
40	TT	CD19+CD27+IgD-CD38 lo/mid	S14000016R	33257	19559	8054
49	II	CD19+CD27+IgD-CD38 lo/mid	S14000021X	25524	18577	6075
50	II	CD19+CD27+IgD-CD38 lo/mid	S14000024A	36904	27741	13736
51	II	CD19+CD27+IgD-CD38 lo/mid	S14000029F	51706	33796	19015
63	II	CD19+CD27+IgD-CD38 lo/mid	S14000035M	33050	18027	8642
64	II	CD19+CD27+IgD-CD38 lo/mid	S14000039R	34019	22131	11603
62	II	CD19+CD27+IgD-CD38 lo/mid	S14000042V	27676	16134	8096
56	II	CD19+CD27+IgD-CD38 lo/mid	S14000063S	66094	47784	15911
59	II	CD19+CD27+IgD-CD38 lo/mid	S14000066W	37125	17060	7657
59	II	CD19+CD27+IgD-CD38 lo/mid	S14000069Z	39425	25384	13482
54	II	CD19+CD27+IgD-CD38 lo/mid	S14000070A	47488	26330	10811
60	II	CD19+CD27+IgD-CD38 lo/mid	S14000084Q	29858	13447	8011
60	II	CD19+CD27+IgD-CD38 lo/mid	S14000091Y	31390	19635	12624
66	II	CD19+CD27+IgD-CD38 lo/mid	S14000093A	43360	24166	10970
72	II	CD19+CD27+IgD-CD38 lo/mid	S14000098F	30913	21542	14656
70	II	CD19+CD27+IgD-CD38 lo/mid	S14000100H	50304	24620	14734
52	II	CD19+CD27+IgD-CD38 lo/mid	S14000112W	32506	22838	11450
52	II	CD19+CD27+IgD-CD38 lo/mid	S14000115Z	46945	29895	16038
53	II	CD19+CD27+IgD-CD38 lo/mid	S14000117B	34868	25761	12935
50	II	CD19+CD27+IgD-CD38 lo/mid	S14000121F	575	447	405
65	TT	CD19+CD27+IgD+	S14000001A	41264	13827	7236
61	TT	CD19+CD27+IgD+	S14000004D	45866	19053	14100
52	TT	CD19+CD27+IgD+	S14000005E	37763	24424	14075
65	TT	CD19+CD27+IgD+	S14000006F	36697	18824	10607
52	TT	CD19+CD27+IgD+	S14000008H	55511	19667	13976
52	TT	CD19+CD27+IgD+	S14000010K	36472	19014	12485
77	TT	CD19+CD27+IgD+	S14000012M	49899	20135	11650
54	TT	CD19+CD27+IgD+	S14000013N	43458	21896	13138
65	TT	CD19+CD27+IgD+	S14000014P	47210	20387	13530
40	TT	CD19+CD27+IgD+	S14000016R	60036	29375	16564
49	II	CD19+CD27+IgD+	S14000021X	42777	22030	12551
51	II	CD19+CD27+IgD+	S14000029F	66026	29935	17194
63	II	CD19+CD27+IgD+	S14000035M	46593	20021	11777
64	II	CD19+CD27+IgD+	S14000039R	41943	16794	9053
62	II	CD19+CD27+IgD+	S14000042V	35364	12651	8789
56	II	CD19+CD27+IgD+	S14000063S	45865	22533	10909
60	II	CD19+CD27+IgD+	S14000084Q	15651	4955	1194
60	II	CD19+CD27+IgD+	S14000091Y	36986	24981	13922
66	II	CD19+CD27+IgD+	S14000093A	55146	33289	18739
72	II	CD19+CD27+IgD+	S14000098F	40510	13626	9770
52	II	CD19+CD27+IgD+	S14000112W	43249	21547	13427
52	II	CD19+CD27+IgD+	S14000115Z	2456	225	110
53	II	CD19+CD27+IgD+	S14000117B	29924	20267	9491
50	II	CD19+CD27+IgD+	S14000121F	43678	25647	15905
65	TT	CD19+IgD-CD27+CD38 hi	S14000001A	48383	32657	11626
61	TT	CD19+IgD-CD27+CD38 hi	S14000004D	40527	22912	6259
52	TT	CD19+IgD-CD27+CD38 hi	S14000005E	31956	18370	6904
52	TT	CD19+IgD-CD27+CD38 hi	S14000008H	41914	21289	5650
52	TT	CD19+IgD-CD27+CD38 hi	S14000010K	51948	29503	11846
77	TT	CD19+IgD-CD27+CD38 hi	S14000012M	42391	24027	8701
54	TT	CD19+IgD-CD27+CD38 hi	S14000013N	20990	13423	5347
65	TT	CD19+IgD-CD27+CD38 hi	S14000014P	35721	13252	4248
40	TT	CD19+IgD-CD27+CD38 hi	S14000016R	42416	25631	10352
49	II	CD19+IgD-CD27+CD38 hi	S14000021X	51233	39433	16904
50	II	CD19+IgD-CD27+CD38 hi	S14000024A	36864	25028	9017
63	II	CD19+IgD-CD27+CD38 hi	S14000035M	50393	35048	15816
64	II	CD19+IgD-CD27+CD38 hi	S14000039R	31080	24881	5869
62	II	CD19+IgD-CD27+CD38 hi	S14000042V	42896	32944	15264
54	II	CD19+IgD-CD27+CD38 hi	S14000070A	63534	33283	5191
66	II	CD19+IgD-CD27+CD38 hi	S14000093A	4078	2129	907
72	II	CD19+IgD-CD27+CD38 hi	S14000098F	41056	27852	9965
70	II	CD19+IgD-CD27+CD38 hi	S14000100H	57094	33841	12401
52	II	CD19+IgD-CD27+CD38 hi	S14000112W	41382	31933	15924
52	II	CD19+IgD-CD27+CD38 hi	S14000115Z	35899	19838	7890
53	II	CD19+IgD-CD27+CD38 hi	S14000117B	28307	16405	4151

50	II	CD19+IgD-CD27+CD38 hi	S14000121F	39707	14464	5869
65	TT	CD19+IgD-CD27-CD38mid/hi	S14000001A	35637	20140	10737
61	TT	CD19+IgD-CD27-CD38mid/hi	S14000004D	44083	26364	12961
52	TT	CD19+IgD-CD27-CD38mid/hi	S14000005E	43894	33919	19287
65	TT	CD19+IgD-CD27-CD38mid/hi	S14000006F	44319	31163	16713
52	TT	CD19+IgD-CD27-CD38mid/hi	S14000010K	45952	21486	11794
77	TT	CD19+IgD-CD27-CD38mid/hi	S14000012M	41460	20471	12206
54	TT	CD19+IgD-CD27-CD38mid/hi	S14000013N	49399	31006	16398
65	TT	CD19+IgD-CD27-CD38mid/hi	S14000014P	27690	10611	6192
49	II	CD19+IgD-CD27-CD38mid/hi	S14000021X	37268	22600	9803
50	II	CD19+IgD-CD27-CD38mid/hi	S14000024A	31012	19979	10405
51	II	CD19+IgD-CD27-CD38mid/hi	S14000029F	36606	29896	16684
63	II	CD19+IgD-CD27-CD38mid/hi	S14000035M	9775	6859	3601
62	II	CD19+IgD-CD27-CD38mid/hi	S14000042V	37236	22683	12649
56	II	CD19+IgD-CD27-CD38mid/hi	S14000063S	4841	694	158
59	II	CD19+IgD-CD27-CD38mid/hi	S14000066W	48443	30508	17571
59	II	CD19+IgD-CD27-CD38mid/hi	S14000069Z	42705	16928	10758
54	II	CD19+IgD-CD27-CD38mid/hi	S14000070A	40753	28137	13751
60	II	CD19+IgD-CD27-CD38mid/hi	S14000084Q	42805	23960	13942
66	II	CD19+IgD-CD27-CD38mid/hi	S14000093A	45494	31339	17682
72	II	CD19+IgD-CD27-CD38mid/hi	S14000098F	35225	14053	8800
70	II	CD19+IgD-CD27-CD38mid/hi	S14000100H	47601	21703	11896
52	II	CD19+IgD-CD27-CD38mid/hi	S14000112W	29983	16067	8869
52	II	CD19+IgD-CD27-CD38mid/hi	S14000115Z	57201	37577	22334
53	II	CD19+IgD-CD27-CD38mid/hi	S14000117B	44446	28015	12767
50	II	CD19+IgD-CD27-CD38mid/hi	S14000121F	31511	20977	12080
65	TT	CD19+IgD+CD27-	S14000001A	35030	13261	9883
61	TT	CD19+IgD+CD27-	S14000004D	38081	13476	10548
52	TT	CD19+IgD+CD27-	S14000005E	31213	12756	8264
65	TT	CD19+IgD+CD27-	S14000006F	22510	5554	3926
52	TT	CD19+IgD+CD27-	S14000008H	43578	13120	9319
52	TT	CD19+IgD+CD27-	S14000010K	27269	6609	5381
77	TT	CD19+IgD+CD27-	S14000012M	56897	18562	13280
54	TT	CD19+IgD+CD27-	S14000013N	21266	5026	3460
50	II	CD19+IgD+CD27-	S14000024A	23913	4967	3256
51	II	CD19+IgD+CD27-	S14000029F	45440	14853	9453
63	II	CD19+IgD+CD27-	S14000035M	36330	12123	8183
64	II	CD19+IgD+CD27-	S14000039R	44436	14925	7690
56	II	CD19+IgD+CD27-	S14000063S	30854	16899	7993
59	II	CD19+IgD+CD27-	S14000066W	43241	11286	7793
59	II	CD19+IgD+CD27-	S14000069Z	48881	19392	14681
54	II	CD19+IgD+CD27-	S14000070A	22839	4859	2513
60	II	CD19+IgD+CD27-	S14000084Q	34825	21492	16160
66	II	CD19+IgD+CD27-	S14000093A	25436	10825	7621
72	II	CD19+IgD+CD27-	S14000098F	35058	15942	12650
52	II	CD19+IgD+CD27-	S14000112W	30784	12757	7500
52	II	CD19+IgD+CD27-	S14000115Z	38028	12227	9350
53	II	CD19+IgD+CD27-	S14000117B	49744	13327	9480
50	II	CD19+IgD+CD27-	S14000121F	30114	14021	10095

Supplementary Table 2: List of antibodies used for mouse flow cytometry

Flow cytometry	Clone	Isotype	Concentration	Catalogue Number	Supplier
Anti-HEL		Rabbit	1/200	discontinued	AbCam
Anti-CD16/32	2.4G2	Rat IgG2b κ	1/500	560541	BD Biosciences
Anti-CD3	145-2C11	Hamster IgG1 κ	1/200	564378	BD Biosciences
Anti-CD4	RM4-5	Rat IgG2a κ	1/300	557956	BD Biosciences
Anti-CD45.1	A20	Mouse IgG2a κ	1/200	561235	BD Biosciences
Anti-CD45.2	104	Mouse IgG2a κ	1/200	45-0454-82	eBioscience
Anti-CD95 / Fas	Jo2	Hamster IgG2 λ2	1/500	557653	BD Biosciences
Anti-GL7	GL7	Rat IgM	1/500	53-5902-82	eBioscience
Anti-Bcl6	K112-91	Mouse IgG1 κ	1/100	561525	BD Biosciences
Anti-CD279 / PD-1	J43	Hamster IgG	1/100	78-9985-82	eBioscience
Anti-Foxp3	FJK-16S	Rat IgG2a κ	1/100	12-5773-82	eBioscience
Anti-CD138 / Syndecan-1	281-2	Rat IgG2a κ	1/400	558626	BD Biosciences
Anti-CD19	1D3	Rat IgG2a κ	1/600	562329	BD Biosciences
Anti-CD45R / B220	RA3-6B2	Rat IgG2a κ	1/400<br>1/600	557957<br>563894	BD Biosciences
Anti-CD93	AA4.1	Rat IgG2b κ	1/100	25-5892-82	eBioscience
Anti-CD21 / CD35	7G6	Rat IgG2b κ	1/200	562756	BD Biosciences
Anti-CD23	B3B4	Rat IgG2a κ	1/200	553139	BD Biosciences
Anti-IgM	II/41	Rat IgG2a κ	1/400	11-5790-81	eBiosciences
Anti-IgD	11-26	Rat IgG2a κ	1/400	12-5993-82	eBiosciences
Streptavidin eFluor450			1/500	48-4317-82	eBiosciences
Anti-ZAP70(pY319)/Syk(pY352)	17A/PZAP70	Mouse IgG1	1/25	557817	BD Biosciences

Supplementary Table 3: Primer list

Primer ID	Primer sequence
IGHA_human_BC	TGTCCAGCACGCTTCAGGCTNNNNTNNNNTNNNNGAYGACCACGTTCCCATCT
IGHM_human_BC	TGTCCAGCACGCTTCAGGCTNNNNTNNNNTNNNNTCGTATCCGACGGGGAATTC
IGHD_human_BC	TGTCCAGCACGCTTCAGGCTNNNNTNNNNTNNNNGGGCTGTTATCCTTTGGGTG
IGHE_human_BC	TGTCCAGCACGCTTCAGGCTNNNNTNNNNTNNNNAGAGTCACGGAGGTGGCATT
IGHG_human_BC	TGTCCAGCACGCTTCAGGCTNNNNTNNNNTNNNNAGTAGTCCTTGACCAGGCAG
VH1-FR2_HUMAN_BIOMED	CTGGGTGCGACAGGCCCCTGGACAA
VH2-FR2_HUMAN_BIOMED	TGGATCCGTCAGCCCCCAGGGAAGG
VH3-FR2_HUMAN_BIOMED	GGTCCGCCAGGCTCCAGGGAA
VH4-FR2_HUMAN_BIOMED	TGGATCCGCCAGCCCCCAGGGAAGG
VH5-FR2_HUMAN_BIOMED	GGGTGCGCCAGATGCCCGGGAAAGG
VH6-FR2_HUMAN_BIOMED	TGGATCAGGCAGTCCCCATCGAGAG
VH7-FR2_HUMAN_BIOMED	TTGGGTGCGACAGGCCCCTGGACAA
CNU Barcode primer_S	TGTCCAGCACGCTTCAGGCT

Supplementary Table 4: Accession numbers for *IGH* sequencing data

Population	Population index
CD19+IgD+CD27-	1
CD19+IgD-CD27-CD38 mid/high	2
CD19+IgD+CD27+	3
CD19+IgD-CD27+CD38 low/mid	4
CD19+IgD-CD27+CD38 hi	5
PBMCs	6

Subject	Genotype	Population index	Sample type	Index	Accession number
S14000001A	TT	1	Flow sorted FCGR2B homozygous healthy individual PBMCs	1	EGAN00001806388
S14000004D	TT	1	Flow sorted FCGR2B homozygous healthy individual PBMCs	2	EGAN00001806385
S14000005E	TT	1	Flow sorted FCGR2B homozygous healthy individual PBMCs	3	EGAN00001806392
S14000006F	TT	1	Flow sorted FCGR2B homozygous healthy individual PBMCs	4	EGAN00001914503
S14000008H	TT	1	Flow sorted FCGR2B homozygous healthy individual PBMCs	5	EGAN00001806380
S14000010K	TT	1	Flow sorted FCGR2B homozygous healthy individual PBMCs	6	EGAN00001914504
S14000012M	TT	1	Flow sorted FCGR2B homozygous healthy individual PBMCs	7	EGAN00001806381
S14000013N	TT	1	Flow sorted FCGR2B homozygous healthy individual PBMCs	8	EGAN00001914505
S14000024A	II	1	Flow sorted FCGR2B homozygous healthy individual PBMCs	11	EGAN00001914506
S14000029F	II	1	Flow sorted FCGR2B homozygous healthy individual PBMCs	12	EGAN00001806393
S14000035M	II	1	Flow sorted FCGR2B homozygous healthy individual PBMCs	13	EGAN00001806387
S14000039R	II	1	Flow sorted FCGR2B homozygous healthy individual PBMCs	14	EGAN00001806389
S14000063S	II	1	Flow sorted FCGR2B homozygous healthy individual PBMCs	16	EGAN00001806379
S14000066W	II	1	Flow sorted FCGR2B homozygous healthy individual PBMCs	17	EGAN00001806386
S14000069Z	II	1	Flow sorted FCGR2B homozygous healthy individual PBMCs	18	EGAN00001806383
S14000070A	II	1	Flow sorted FCGR2B homozygous healthy individual PBMCs	19	EGAN00001914509
S14000084Q	II	1	Flow sorted FCGR2B homozygous healthy individual PBMCs	20	EGAN00001806384
S14000093A	II	1	Flow sorted FCGR2B homozygous healthy individual PBMCs	21	EGAN00001806390
S14000098F	II	1	Flow sorted FCGR2B homozygous healthy individual PBMCs	22	EGAN00001806382
S14000112W	II	1	Flow sorted FCGR2B homozygous healthy individual PBMCs	23	EGAN00001806394
S14000115Z	II	1	Flow sorted FCGR2B homozygous healthy individual PBMCs	24	EGAN00001806395

S14000117B	II	1	Flow sorted FCGR2B homozygous healthy individual PBMCs	25	EGAN00001806396
S14000121F	II	1	Flow sorted FCGR2B homozygous healthy individual PBMCs	26	EGAN00001806391
S14000001A	TT	2	Flow sorted FCGR2B homozygous healthy individual PBMCs	27	EGAN00001806315
S14000004D	TT	2	Flow sorted FCGR2B homozygous healthy individual PBMCs	28	EGAN00001806313
S14000005E	TT	2	Flow sorted FCGR2B homozygous healthy individual PBMCs	29	EGAN00001806321
S14000006F	TT	2	Flow sorted FCGR2B homozygous healthy individual PBMCs	30	EGAN00001806319
S14000010K	TT	2	Flow sorted FCGR2B homozygous healthy individual PBMCs	31	EGAN00001806327
S14000012M	TT	2	Flow sorted FCGR2B homozygous healthy individual PBMCs	32	EGAN00001806307
S14000013N	TT	2	Flow sorted FCGR2B homozygous healthy individual PBMCs	33	EGAN00001806328
S14000014P	TT	2	Flow sorted FCGR2B homozygous healthy individual PBMCs	34	EGAN00001806310
S14000021X	II	2	Flow sorted FCGR2B homozygous healthy individual PBMCs	35	EGAN00001806323
S14000024A	II	2	Flow sorted FCGR2B homozygous healthy individual PBMCs	36	EGAN00001806322
S14000029F	II	2	Flow sorted FCGR2B homozygous healthy individual PBMCs	37	EGAN00001806324
S14000035M	II	2	Flow sorted FCGR2B homozygous healthy individual PBMCs	38	EGAN00001914507
S14000042V	II	2	Flow sorted FCGR2B homozygous healthy individual PBMCs	39	EGAN00001806316
S14000063S	II	2	Flow sorted FCGR2B homozygous healthy individual PBMCs	40	EGAN00001914508
S14000066W	II	2	Flow sorted FCGR2B homozygous healthy individual PBMCs	41	EGAN00001806314
S14000069Z	II	2	Flow sorted FCGR2B homozygous healthy individual PBMCs	42	EGAN00001806311
S14000070A	II	2	Flow sorted FCGR2B homozygous healthy individual PBMCs	43	EGAN00001806306
S14000084Q	II	2	Flow sorted FCGR2B homozygous healthy individual PBMCs	44	EGAN00001806312
S14000091Y	II	2	Flow sorted FCGR2B homozygous healthy individual PBMCs	45	EGAN00001806317
S14000093A	II	2	Flow sorted FCGR2B homozygous healthy individual PBMCs	46	EGAN00001806318
S14000098F	II	2	Flow sorted FCGR2B homozygous healthy individual PBMCs	47	EGAN00001806308
S14000100H	II	2	Flow sorted FCGR2B homozygous healthy individual PBMCs	48	EGAN00001806309
S14000112W	II	2	Flow sorted FCGR2B homozygous healthy individual PBMCs	49	EGAN00001806325
S14000115Z	II	2	Flow sorted FCGR2B homozygous healthy individual PBMCs	50	EGAN00001806326
S14000117B	II	2	Flow sorted FCGR2B homozygous healthy individual PBMCs	51	EGAN00001806329
S14000121F	II	2	Flow sorted FCGR2B homozygous healthy individual PBMCs	52	EGAN00001806320

S14000001A	TT	3	Flow sorted FCGR2B homozygous healthy individual PBMCs	53	EGAN00001806291
S14000004D	TT	3	Flow sorted FCGR2B homozygous healthy individual PBMCs	54	EGAN00001806289
S14000005E	TT	3	Flow sorted FCGR2B homozygous healthy individual PBMCs	55	EGAN00001806298
S14000006F	TT	3	Flow sorted FCGR2B homozygous healthy individual PBMCs	56	EGAN00001806296
S14000008H	TT	3	Flow sorted FCGR2B homozygous healthy individual PBMCs	57	EGAN00001806285
S14000010K	TT	3	Flow sorted FCGR2B homozygous healthy individual PBMCs	58	EGAN00001806303
S14000012M	TT	3	Flow sorted FCGR2B homozygous healthy individual PBMCs	59	EGAN00001806286
S14000013N	TT	3	Flow sorted FCGR2B homozygous healthy individual PBMCs	60	EGAN00001806304
S14000014P	TT	3	Flow sorted FCGR2B homozygous healthy individual PBMCs	61	EGAN00001806288
S14000016R	TT	3	Flow sorted FCGR2B homozygous healthy individual PBMCs	62	EGAN00001806299
S14000021X	II	3	Flow sorted FCGR2B homozygous healthy individual PBMCs	63	EGAN00001806300
S14000029F	II	3	Flow sorted FCGR2B homozygous healthy individual PBMCs	64	EGAN00001806301
S14000035M	II	3	Flow sorted FCGR2B homozygous healthy individual PBMCs	65	EGAN00001806290
S14000039R	II	3	Flow sorted FCGR2B homozygous healthy individual PBMCs	66	EGAN00001806292
S14000042V	II	3	Flow sorted FCGR2B homozygous healthy individual PBMCs	67	EGAN00001806293
S14000063S	II	3	Flow sorted FCGR2B homozygous healthy individual PBMCs	68	EGAN00001806284
S14000084Q	II	3	Flow sorted FCGR2B homozygous healthy individual PBMCs	69	EGAN00001914510
S14000091Y	II	3	Flow sorted FCGR2B homozygous healthy individual PBMCs	70	EGAN00001806294
S14000093A	II	3	Flow sorted FCGR2B homozygous healthy individual PBMCs	71	EGAN00001806295
S14000098F	II	3	Flow sorted FCGR2B homozygous healthy individual PBMCs	72	EGAN00001806287
S14000112W	II	3	Flow sorted FCGR2B homozygous healthy individual PBMCs	73	EGAN00001806302
S14000117B	II	3	Flow sorted FCGR2B homozygous healthy individual PBMCs	75	EGAN00001806305
S14000121F	II	3	Flow sorted FCGR2B homozygous healthy individual PBMCs	76	EGAN00001806297
S14000001A	TT	4	Flow sorted FCGR2B homozygous healthy individual PBMCs	77	EGAN00001806342
S14000004D	TT	4	Flow sorted FCGR2B homozygous healthy individual PBMCs	78	EGAN00001806339
S14000005E	TT	4	Flow sorted FCGR2B homozygous healthy individual PBMCs	79	EGAN00001806348
S14000006F	TT	4	Flow sorted FCGR2B homozygous healthy individual PBMCs	80	EGAN00001806347
S14000008H	TT	4	Flow sorted FCGR2B homozygous healthy individual PBMCs	81	EGAN00001806332

S14000010K	TT	4	Flow sorted FCGR2B homozygous healthy individual PBMCs	82	EGAN00001806355
S14000012M	TT	4	Flow sorted FCGR2B homozygous healthy individual PBMCs	83	EGAN00001806333
S14000013N	TT	4	Flow sorted FCGR2B homozygous healthy individual PBMCs	84	EGAN00001806356
S14000014P	TT	4	Flow sorted FCGR2B homozygous healthy individual PBMCs	85	EGAN00001806336
S14000016R	TT	4	Flow sorted FCGR2B homozygous healthy individual PBMCs	86	EGAN00001806350
S14000021X	II	4	Flow sorted FCGR2B homozygous healthy individual PBMCs	87	EGAN00001806351
S14000024A	II	4	Flow sorted FCGR2B homozygous healthy individual PBMCs	88	EGAN00001806349
S14000029F	II	4	Flow sorted FCGR2B homozygous healthy individual PBMCs	89	EGAN00001806352
S14000035M	II	4	Flow sorted FCGR2B homozygous healthy individual PBMCs	90	EGAN00001806341
S14000039R	II	4	Flow sorted FCGR2B homozygous healthy individual PBMCs	91	EGAN00001806343
S14000042V	II	4	Flow sorted FCGR2B homozygous healthy individual PBMCs	92	EGAN00001806344
S14000063S	II	4	Flow sorted FCGR2B homozygous healthy individual PBMCs	93	EGAN00001806330
S14000066W	II	4	Flow sorted FCGR2B homozygous healthy individual PBMCs	94	EGAN00001806340
S14000069Z	II	4	Flow sorted FCGR2B homozygous healthy individual PBMCs	95	EGAN00001806337
S14000070A	II	4	Flow sorted FCGR2B homozygous healthy individual PBMCs	96	EGAN00001806331
S14000084Q	II	4	Flow sorted FCGR2B homozygous healthy individual PBMCs	97	EGAN00001806338
S14000091Y	II	4	Flow sorted FCGR2B homozygous healthy individual PBMCs	98	EGAN00001806345
S14000093A	II	4	Flow sorted FCGR2B homozygous healthy individual PBMCs	99	EGAN00001806346
S14000098F	II	4	Flow sorted FCGR2B homozygous healthy individual PBMCs	100	EGAN00001806334
S14000100H	II	4	Flow sorted FCGR2B homozygous healthy individual PBMCs	101	EGAN00001806335
S14000112W	II	4	Flow sorted FCGR2B homozygous healthy individual PBMCs	102	EGAN00001806353
S14000115Z	II	4	Flow sorted FCGR2B homozygous healthy individual PBMCs	103	EGAN00001806354
S14000117B	II	4	Flow sorted FCGR2B homozygous healthy individual PBMCs	104	EGAN00001806357
S14000121F	II	4	Flow sorted FCGR2B homozygous healthy individual PBMCs	105	EGAN00001914512
S14000001A	TT	5	Flow sorted FCGR2B homozygous healthy individual PBMCs	106	EGAN00001806366
S14000004D	TT	5	Flow sorted FCGR2B homozygous healthy individual PBMCs	107	EGAN00001806364
S14000005E	TT	5	Flow sorted FCGR2B homozygous healthy individual PBMCs	108	EGAN00001806370
S14000008H	TT	5	Flow sorted FCGR2B homozygous healthy individual PBMCs	109	EGAN00001806359

S14000010K	TT	5	Flow sorted FCGR2B homozygous healthy individual PBMCs	110	EGAN00001806376
S14000012M	TT	5	Flow sorted FCGR2B homozygous healthy individual PBMCs	111	EGAN00001806360
S14000013N	TT	5	Flow sorted FCGR2B homozygous healthy individual PBMCs	112	EGAN00001806377
S14000014P	TT	5	Flow sorted FCGR2B homozygous healthy individual PBMCs	113	EGAN00001806363
S14000016R	TT	5	Flow sorted FCGR2B homozygous healthy individual PBMCs	114	EGAN00001806372
S14000021X	II	5	Flow sorted FCGR2B homozygous healthy individual PBMCs	115	EGAN00001806373
S14000024A	II	5	Flow sorted FCGR2B homozygous healthy individual PBMCs	116	EGAN00001806371
S14000035M	II	5	Flow sorted FCGR2B homozygous healthy individual PBMCs	117	EGAN00001806365
S14000039R	II	5	Flow sorted FCGR2B homozygous healthy individual PBMCs	118	EGAN00001806367
S14000042V	II	5	Flow sorted FCGR2B homozygous healthy individual PBMCs	119	EGAN00001806368
S14000070A	II	5	Flow sorted FCGR2B homozygous healthy individual PBMCs	121	EGAN00001806358
S14000093A	II	5	Flow sorted FCGR2B homozygous healthy individual PBMCs	122	EGAN00001914511
S14000098F	II	5	Flow sorted FCGR2B homozygous healthy individual PBMCs	123	EGAN00001806361
S14000100H	II	5	Flow sorted FCGR2B homozygous healthy individual PBMCs	124	EGAN00001806362
S14000112W	II	5	Flow sorted FCGR2B homozygous healthy individual PBMCs	125	EGAN00001806374
S14000115Z	II	5	Flow sorted FCGR2B homozygous healthy individual PBMCs	126	EGAN00001806375
S14000117B	II	5	Flow sorted FCGR2B homozygous healthy individual PBMCs	127	EGAN00001806378
S14000121F	II	5	Flow sorted FCGR2B homozygous healthy individual PBMCs	128	EGAN00001806369
C11	Healthy	6	Total PBMC BCR sequencing	129	EGAN00001588633
C7	Healthy	6	Total PBMC BCR sequencing	130	EGAN00001588631
C25	Healthy	6	Total PBMC BCR sequencing	131	EGAN00001588636
C28	Healthy	6	Total PBMC BCR sequencing	132	EGAN00001588634
C30	Healthy	6	Total PBMC BCR sequencing	133	EGAN00001588635
C4	Healthy	6	Total PBMC BCR sequencing	134	EGAN00001588633
C502	Healthy	6	Total PBMC BCR sequencing	135	EGAN00001588625
C504	Healthy	6	Total PBMC BCR sequencing	136	EGAN00001588728
C13	Healthy	6	Total PBMC BCR sequencing	137	EGAN00001588630
C14	Healthy	6	Total PBMC BCR sequencing	138	EGAN00001588717
C15	Healthy	6	Total PBMC BCR sequencing	139	EGAN00001588725
C20	Healthy	6	Total PBMC BCR sequencing	140	EGAN00001588611
C21	Healthy	6	Total PBMC BCR sequencing	141	EGAN00001588632
C22	Healthy	6	Total PBMC BCR sequencing	142	EGAN00001588634
C23	Healthy	6	Total PBMC BCR sequencing	143	EGAN00001588631
C505	Healthy	6	Total PBMC BCR sequencing	144	EGAN00001588635
C507	Healthy	6	Total PBMC BCR sequencing	145	EGAN00001588636
C509	Healthy	6	Total PBMC BCR sequencing	146	EGAN00001588624
C510	Healthy	6	Total PBMC BCR sequencing	147	EGAN00001588736

C513	Healthy	6	Total PBMC BCR sequencing	148	EGAN00001588744
C514	Healthy	6	Total PBMC BCR sequencing	149	EGAN00001588752
C516	Healthy	6	Total PBMC BCR sequencing	150	EGAN00001588760
C517	Healthy	6	Total PBMC BCR sequencing	151	EGAN00001588614
C520	Healthy	6	Total PBMC BCR sequencing	152	EGAN00001588776
C521	Healthy	6	Total PBMC BCR sequencing	153	EGAN00001588697
C525	Healthy	6	Total PBMC BCR sequencing	154	EGAN00001588705
C527	Healthy	6	Total PBMC BCR sequencing	155	EGAN00001588629
C8	Healthy	6	Total PBMC BCR sequencing	156	EGAN00001588629
C17	Healthy	6	Total PBMC BCR sequencing	157	EGAN00001588630
58	SLE	6	Total PBMC BCR sequencing	158	EGAN00001588598
183	SLE	6	Total PBMC BCR sequencing	159	EGAN00001588672
241	SLE	6	Total PBMC BCR sequencing	160	EGAN00001806411
251	SLE	6	Total PBMC BCR sequencing	161	EGAN00001806412
256	SLE	6	Total PBMC BCR sequencing	162	EGAN00001806413
260	SLE	6	Total PBMC BCR sequencing	163	EGAN00001806414
301	SLE	6	Total PBMC BCR sequencing	164	EGAN00001806415
302	SLE	6	Total PBMC BCR sequencing	165	EGAN00001806416
303	SLE	6	Total PBMC BCR sequencing	166	EGAN00001806417
