# Supplementary material for: FcγRIIb differentially regulates pre-immune and germinal center B cell tolerance in mouse and human
Source: Nat Commun. 2019 Apr 29;10:1970. doi: 10.1038/s41467-019-09434-0 (PMC6488660; doi:10.1038/s41467-019-09434-0)
Supplement: Supplementary file 3 — Reporting Summary [file 41467_2019_9434_MOESM3_ESM.pdf]

nature research

Corresponding author(s): *Double-blind peer review submissions: write DBPR and your manuscript number here instead of author names.*

Reporting Summary

Nature Research wishes to improve the reproducibility of the work that we publish. This form provides structure for consistency and transparency in reporting. For further information on Nature Research policies, see Authors & Referees and the Editorial Policy Checklist.

Statistical parameters

When statistical analyses are reported, confirm that the following items are present in the relevant location (e.g. figure legend, table legend, main text, or Methods section).

n/a	Confirmed	
☐	☒	The exact sample size (n) for each experimental group/condition, given as a discrete number and unit of measurement
☐	☒	An indication of whether measurements were taken from distinct samples or whether the same sample was measured repeatedly
☐	☒	The statistical test(s) used AND whether they are one- or two-sided<br>*Only common tests should be described solely by name; describe more complex techniques in the Methods section.*
☐	☒	A description of all covariates tested
☒	☐	A description of any assumptions or corrections, such as tests of normality and adjustment for multiple comparisons
☐	☒	A full description of the statistics including central tendency (e.g. means) or other basic estimates (e.g. regression coefficient) AND variation (e.g. standard deviation) or associated estimates of uncertainty (e.g. confidence intervals)
☒	☐	For null hypothesis testing, the test statistic (e.g. F, t, r) with confidence intervals, effect sizes, degrees of freedom and P value noted<br>*Give P values as exact values whenever suitable.*
☒	☐	For Bayesian analysis, information on the choice of priors and Markov chain Monte Carlo settings
☒	☐	For hierarchical and complex designs, identification of the appropriate level for tests and full reporting of outcomes
☒	☐	Estimates of effect sizes (e.g. Cohen's d, Pearson's r), indicating how they were calculated
☐	☒	Clearly defined error bars<br>*State explicitly what error bars represent (e.g. SD, SE, CI)*

Our web collection on statistics for biologists may be useful.

Software and code

Policy information about availability of computer code

Data collection	Flow cytometry data ware collected using the Diva software (Becton Dickinson).
Data analysis	Mouse data were analysed using Flowjo, Excel and Prism. Human sequencing data were analysed using QUASR, BLAST, IMGT V-QUEST, Immune_receptor_NETWORK-GENERATION and R. The source and location of all softwares are described in the material and methods.

For manuscripts utilizing custom algorithms or software that are central to the research but not yet described in published literature, software must be made available to editors/reviewers upon request. We strongly encourage code deposition in a community repository (e.g. GitHub). See the Nature Research guidelines for submitting code & software for further information.

Data

Policy information about availability of data

All manuscripts must include a data availability statement. This statement should provide the following information, where applicable:

- Accession codes, unique identifiers, or web links for publicly available datasets
- A list of figures that have associated raw data
- A description of any restrictions on data availability

A reporting summary for this article is available as a Supplementary Information file. The source data underlying Figs 1 to 6 and Figs S1 to S6 are available as a Source Data file. EGA accession numbers for sequencing data are provided in Table S4.

Field-specific reporting

Please select the best fit for your research. If you are not sure, read the appropriate sections before making your selection.

☒ Life sciences ☐ Behavioural & social sciences ☐ Ecological, evolutionary & environmental sciences

For a reference copy of the document with all sections, see nature.com/authors/policies/ReportingSummary-flat.pdf

Life sciences study design

All studies must disclose on these points even when the disclosure is negative.

Sample size	the number of animals included in each experiments was determined based on our previous expertise using mouse models with different expression level of FcγRIIb and by using the G*Power software for a priori power calculation.
Data exclusions	For bone marrow chimera experiments, mice that were not fully reconstituted (% of B cells <10%) were excluded. For immunization experiments, mice that were not properly immunized (total germinal centre B cells <1% of total B cells) were excluded.
Replication	All experiments were replicated at least twice and gave similar results in each replicate.
Randomization	no randomization was used
Blinding	After irradiation and reconstitution animals were numbered independently of their genotype or of the bone marrow they received before the immunization. The number was linked to the genotype and bone marrow injected only after the analysis was done.

Reporting for specific materials, systems and methods

Materials & experimental systems

n/a	Involved in the study
☒	☐ Unique biological materials
☐	☒ Antibodies
☒	☐ Eukaryotic cell lines
☒	☐ Palaeontology
☐	☒ Animals and other organisms
☐	☒ Human research participants

Methods

n/a	Involved in the study
☒	☐ ChIP-seq
☐	☒ Flow cytometry
☒	☐ MRI-based neuroimaging

Antibodies

Antibodies used	All the clone numbers, dilutions and reference numbers are indicated in the methods and Table S3 .
Validation	*Describe the validation of each primary antibody for the species and application, noting any validation statements on the manufacturer's website, relevant citations, antibody profiles in online databases, or data provided in the manuscript.*

Animals and other organisms

Policy information about studies involving animals; ARRIVE guidelines recommended for reporting animal research

Laboratory animals	C57BL/6 male and females between 10-20 weeks see methods
Wild animals	*Provide details on animals observed in or captured in the field; report species, sex and age where possible. Describe how animals were caught and transported and what happened to captive animals after the study (if killed, explain why and describe method; if released, say where and when) OR state that the study did not involve wild animals.*
Field-collected samples	*For laboratory work with field-collected samples, describe all relevant parameters such as housing, maintenance, temperature, photoperiod and end-of-experiment protocol OR state that the study did not involve samples collected from the field.*

Human research participants

Policy information about studies involving human research participants

Population characteristics	The population characteristics are described in the methods and in Tables S1
Recruitment	The recruitment strategy is described in the methods

Flow Cytometry

Plots

Confirm that:

- [x] The axis labels state the marker and fluorochrome used (e.g. CD4-FITC).
- [x] The axis scales are clearly visible. Include numbers along axes only for bottom left plot of group (a 'group' is an analysis of identical markers).
- [x] All plots are contour plots with outliers or pseudocolor plots.
- [x] A numerical value for number of cells or percentage (with statistics) is provided.

Methodology

Sample preparation	Mouse spleens were passed through a cell strainer with culture medium. Mouse bone marrow was flushed out of the bone with culture medium. Red blood cells were lyzed from both single cell suspensions with ACK buffer.
Instrument	FACS analysis was performed on a BD LSR Fortessa (BD biosciences)
Software	data were analyzed with the Flowjo software (TreeStar, Ashland, OR)
Cell population abundance	Purity was determined by post-sort flow-based purity check (>95%) and through assessment of isotype usages within the sorted populations from the sequencing data.
Gating strategy	Cells were gated on a FSC/SSC dot plot, singlets were excluded and live cells were gated as the negative population on a SSC/live-dead staining. All following gating strategies are representated in the paper.

- [x] Tick this box to confirm that a figure exemplifying the gating strategy is provided in the Supplementary Information.
